# Anatomic versus non‐anatomic liver resection for hepatocellular carcinoma—A European multicentre cohort study in cirrhotic and non‐cirrhotic patients

**DOI:** 10.1002/cam4.6981

**Published:** 2024-03-13

**Authors:** Jasmin Zeindler, Gabriel Fridolin Hess, Maximilian von Heesen, Noa Aegerter, Cornelia Reber, Andreas Michael Schmitt, Simone Muenst, Martin Bolli, Savas Deniz Soysal, Otto Kollmar

**Affiliations:** ^1^ Clarunis University Centre for Gastrointestinal and Liver Diseases Basel Switzerland; ^2^ Department of General, Visceral, Vascular and Pediatric Surgery University of Saarland Homburg/Saar Germany; ^3^ Department of General‐ and Visceral Surgery University Hospital Göttingen Göttingen Germany; ^4^ Faculty of Medicine University of Basel Basel Switzerland; ^5^ The Royal Marsden NHS Foundation Trust London UK; ^6^ Department of Medical Oncology University Hospital Basel Basel Switzerland; ^7^ Institute of Medical Genetics and Pathology University Hospital Basel Basel Switzerland

**Keywords:** anatomic liver resection, cirrhosis, hepatocellular carcinoma, liver resection, non‐anatomic liver resection, surgical oncology

## Abstract

**Background:**

The incidence of hepatocellular carcinoma (HCC) is increasing in the western world over the past decades. As liver resection (LR) represents one of the most efficient treatment options, advantages of anatomic (ALR) versus non‐anatomic liver resection (NALR) show a lack of consistent evidence. Therefore, the aim of this study was to investigate complications and survival rates after both resection types.

**Methods:**

This is a multicentre cohort study using retrospectively and prospectively collected data. We included all patients undergoing LR for HCC between 2009 and 2020 from three specialised centres in Switzerland and Germany. Complication and survival rates after ALR versus NALR were analysed using uni‐ and multivariate Cox regression models.

**Results:**

Two hundred and ninety‐eight patients were included. Median follow‐up time was 52.76 months. 164/298 patients (55%) underwent ALR. Significantly more patients with cirrhosis received NALR (*n* = 94/134; *p* < 0.001). Complications according to the Clavien Dindo classification were significantly more frequent in the NALR group (*p* < 0.001). Liver failure occurred in 13% after ALR versus 8% after NALR (*p* < 0.215). Uni‐ and multivariate cox regression models showed no significant differences between the groups for recurrence free survival (RFS) and overall survival (OS). Furthermore, cirrhosis had no significant impact on OS and RFS.

**Conclusion:**

No significant differences on RFS and OS rates could be observed. Post‐operative complications were significantly less frequent in the ALR group while liver specific complications were comparable between both groups. Subgroup analysis showed no significant influence of cirrhosis on the post‐operative outcome of these patients.

## Graphical abstract

Investigation of Anatomic (ALR) versus non‐anatomic liver resection for hepatocellular carcinoma showed no significant differences on recurrence free survival and overall survival rates. Post‐operative complications were significantly less frequent in the ALR group while liver specific complications were comparable between both groups. Subgroup analysis showed no significant influence of cirrhosis on the post‐operative outcome of these patients.

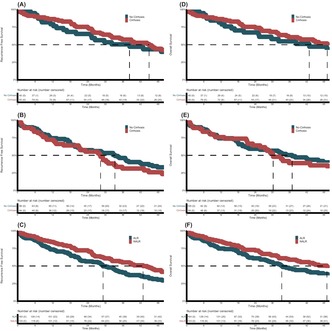

## 1 INTRODUCTION

Hepatocellular carcinoma (HCC) is the second leading cause of cancer‐associated deaths globally^1^, ^2^ and the most common primary liver malignancy.^3^ In the past few decades, the incidence of HCC has been rising in the Western World.^4^ HCCs usually occur within an underlying chronic liver disease such as liver cirrhosis with limited liver function and therefore it is associated with a short survival time after diagnosis without treatment.^5^, ^6^ Prognosis is determined not only by the tumour burden, but also the remaining liver function. Five‐year survival rates are around 60% in high‐volume centres.^7^ The most effective therapies include liver resection (LR) and liver transplantation,^8^, ^9^, ^10^ with LR being the most widely advised first‐line therapy.^11^, ^12^, ^13^ Due to advances in surgical techniques and perioperative care, LR clearly improves the outcome with low perioperative morbidity and mortality.^11^, ^14^, ^15^ Currently, 30‐day mortality after LR for HCC is below 5% in specialised centres. The extent of LR and the cirrhotic status influence the direct outcome of patients undergoing LR for HCC as preserved liver parenchyma prevents post‐operative liver failure and higher extent of resection is associated with a worse post‐operative outcome.^16^ Furthermore, post‐operative complications are known to be associated with poorer oncological outcome.^17^, ^18^, ^19^ Unfortunately, as HCC is a primary liver tumour within a diseased liver, tumour recurrence of HCC after LR remain still high, as shown by more than 50% of the patients with loco‐regional recurrence within 5 years after resection.^7^

In 1985, Makuuchi et al. first described the technique of anatomic LR (ALR), defined as tumour resection in to with the corresponding liver segment or subsegment, including tumour‐bearing portal tributaries, and one major branch of the hepatic artery and the portal vein.^20^, ^21^ In contrast, non‐anatomic LR (NALR) aims to spare hepatic parenchyma, is therefore less extensive, and defined as limited resection or enucleation regardless the underlying hepatic anatomy. Therefore, this technique reduces the risk of perioperative liver failure, especially in cirrhotic patients.^22^

The surgical challenges of LR are preventing recurrences with post‐operative tumour‐free resection margins while preserving sufficient liver parenchyma to avoid post‐operative hepatic failure. Currently, a controversy regarding the benefits of ALR versus NALR for HCC patients exists among experts, with a lack of consistent evidence favouring one resection technique above the other especially within the Western World.^22^ The current literature does not show any differences in perioperative complications, overall survival (OS) and recurrence free survival (RFS) comparing ALR and NALR for HCC patients.^22^, ^23^ Studies directly comparing both surgical methods are limited, especially within the Western World. Therefore, we conducted a European multicentre study to investigate short‐term and long‐term complications as well as oncological outcome after ALR versus NALR in cirrhotic and non‐cirrhotic patients with HCC.

## 2 METHODS

### 2.1 Study design and data collection

This is a cohort study using retrospectively and prospectively collected data. Reporting follows the Strengthening the Reporting of Observational Studies in Epidemiology (STROBE) guidelines when applicable.^24^ The study was approved by the local ethics committee (ref. no EK 316/12) and conducted according to the Swiss federal act on research involving human beings (Human Research Act) and the guidelines of good clinical practice. We included all patients undergoing LR for HCC between 2009 and 2020 from three specialised centres in Switzerland and Germany (University Hospital Basel, St. Claraspital Basel and University Hospital Homburg/Saar).

All data from Homburg/Saar were prospectively entered in an ISH‐Med (GSD, Berlin, Germany) database running on a SAP platform (SAP, St. Leon‐Roth, Germany), whereas data from both Basel centres were collected retrospectively before 2019, and prospectively after. All data were handled anonymously and recorded in an Excel spread sheet with a unique patient identifier. Collected variables included patient‐related, tumour‐related and treatment‐related data. Post‐operative complications were recorded according to the Clavien‐Dindo Classification.^25^ ALR were defined by the Brisbane 2000 nomenclature of liver anatomy and reception.^26^ NALR included wedge resections or more limited resections. Posthepatectomy liver failure was defined according to the consensus of the International Study Group of Liver Surgeries (ISGLS) using laboratory and clinical parameters.^27^

All cases were discussed at the interdisciplinary tumour board. Surgical resection was performed by an experienced HPB team. All three institutions followed the current guidelines of treatment and standard of care.^28^ All patients were followed up according to surveillance guidelines in Germany and Switzerland for HCC, involving regular measurement of serum AFP levels and imaging. Diagnosis of recurrence was defined as classical arterial contrast enhancement and venous wash out in CT scans or MRI or histologically confirmed cancer recurrence. The date of the last follow up as well as date of recurrence and death was registered.

### 2.2 Statistical analysis

We stratified patients according to the type of resection. Patient, tumour and treatment characteristics were analysed for all patients and compared between the two groups using descriptive statistics.

OS was defined as the time between the date of surgery and death due to any cause. RFS was defined as the time between the date of surgery and the date of recurrence or death, whatever occurred first. OS and RFS were visualised using the Kaplan–Meier method. We used a Cox regression model to calculate the corresponding hazard ratio (HR) for OS and RFS. In an additional multivariable analysis, we adjusted for the following variables: Sex, centre, stage, BMI, ASA classification, cirrhosis, portal hypertension, diabetes mellitus, cardiac comorbidities, tumour size and type of resection. The follow up time for the entire cohort was estimated using the inverse Kaplan–Meier estimator. We used R for data cleaning and analyses (The R Project for Statistical Computing, https://www.r‐project.org/).

## 3 RESULTS

### 3.1 Baseline characteristics

A total of 298 patients were included in this analysis. 55% underwent ALR, 45% underwent NALR. Patients undergoing ALR received in 24% one segmentectomy (40/164), in 32% more than one segmentectomy (52/164) and in 38% hemihepatectomies (62/164). In 10 cases (6%), data of the type of ALR were missing. The majority of resections (59%) were performed in Homburg/Saar, 66% of these patients underwent ALR. Cirrhosis was present in 53% of patients. 59.4% of patients with liver cirrhosis received NALR. The most common cause of cirrhosis was alcohol (30%) and HCV (25%). UICC cancer stage was significantly different between the two treatment groups (*p* < 0.001). The majority of patients (91%) undergoing NALR showed UICC stage I or II. Significantly more patients with liver cirrhosis received NALR (*p* < 0.001). Furthermore, portal hypertension was also significantly more present in the NALR group (*p* < 0.001). For more details see Table 1.

**TABLE 1:** Baseline characteristics.

	All patients	ALR	NALR	*p*‐value
	*N* = 298	*N* = 164	*N* = 134	
Sex; male, *n* (%)	220 (74)	123 (75)	97 (72)	0.71
Age; years, mean (SD)	67 (11)	67 (11)	67 (11)	0.52
Center, *n* (%)				
St. Clara Spital	13 (4)	7 (4)	6 (4)	0.03
University Homburg/Saar	176 (59)	108 (66)	68 (51)	
University Basel	109 (37)	49 (30)	60 (45)	
BMI–mean (SD)	27 (5)	26 (4)	28 (5)	<0.01
Stage, *n* (%)				
I	139 (47)	61 (38)	78 (59)	<0.01
II	86 (29)	44 (27)	42 (32)	
III	56 (19)	46 (29)	10 (8)	
IV	13 (4)	10 (6)	3 (2)	
Cirrhosis, *n* (%)	158 (53)	64 (39)	94 (70)	<0.01
Cause of cirrhosis				
Auto immune	2 (1)	1 (2)	1 (1)	<0.01
Hemochromatosis	5 (3)	0 (0)	5 (5)	
Ethyltoxic	48 (30)	13 (20)	35 (37)	
HBV	18 (11)	10 (16)	8 (9)	
HCV	39 (25)	12 (19)	27 (29)	
Mixed type	12 (8)	4 (6)	8 (9)	
Unknown	34 (22)	24 (38)	10 (11)	
Child‐Pugh Score				
A	121 (77)	48 (75)	73 (78)	0.38
B	24 (15)	9 (14)	15 (16)	
C	4 (3)	1 (2)	3 (3)	
Unknown	9 (6)	6 (9)	3 (3)	
ASA, *n* (%)				
2	91 (33)	54 (35)	37 (30)	0.12
3	176 (64)	98 (64)	78 (63)	
4	10 (3)	2 (1)	8 (6)	
Portal hypertension, *n* (%)	53 (18)	17 (10)	36 (27)	<0.001
Diabetes mellitus, *n* (%)	124 (42)	67 (41)	57 (43)	0.86
Cardiac disease, *n* (%)	66 (22)	44 (27)	22 (16)	0.04
Neurologic disease, *n* (%)	57 (19)	30 (18)	27 (20)	0.80

Abbreviations: ALR, anatomic liver resection; NALR, non‐anatomic liver resection.

### 3.2 Complications according to the type of liver resection

Details regarding morbidity and type of LR are shown in Table 2. Complications according to the Clavien Dindo classification were significantly more frequent after NARL (*p* < 0.001). Only 5% of patients died. The majority of patients suffered from mild post‐operative complications grade I. Severe complications (grade III and IV) were more frequent after NALR (14% vs. 10%, and 23% vs. 5%; respectively). Specific complications after liver surgery were not significantly different. Post‐operative liver insufficiency was detected in 13% after ALR versus 8% after NALR (*p* = 0.22). There was no significant difference regarding complications comparing patients with and without cirrhosis (*p* = 0.07). Cirrhotic patients did not develop more often post‐operative liver insufficiency (*p* = 0.46).

**TABLE 2:** Details surgery and morbidity.

	All patients	ALR	NALR	*p*‐value	Cirrhosis	No Cirrhosis	*p*‐value
	*N* = 298	*N* = 164	*N* = 134		*N* = 158	*N* = 140	
Days on ICU, mean (SD)	4 (19)	5 (21)	3 (14)	0.469	3 (5)	5 (26)	0.29
Post‐operative days, mean (SD)	15 (21)	16 (23)	13 (18)	0.170	13 (9)	17 (29)	0.16
Complications (Clavien Dindo), *n* (%)							
0	129 (43)	111 (68)	18 (14)	< 0.001	56 (36)	73 (52)	0.07
1	37 (12)	12 (7)	25 (19)		24 (15)	13 (9)	
2	43 (14)	11 (7)	32 (24)		24 (15)	19 (14)	
3	35 (12)	16 (10)	19 (14)		20 (13)	15 (11)	
4	38 (13)	8 (5)	30 (23)		22 (14)	16 (11)	
5	15 (5)	6 (4)	9 (7)		11 (7)	4 (3)	
Specific post‐operative complications, *n* (%)							
Bilioma	19 (6)	15 (9)	4 (3)	0.054	9 (6)	10 (6)	1.00
Bleeding	21 (7)	10 (6)	11 (8)	0.631	10 (7)	11 (7)	1.00
Liver abscess	5 (2)	5 (3)	0 (0)	0.113	1 (1)	4 (3)	0.44
Liver insufficiency	33 (11)	22 (13)	11 (8)	0.215	13 (9)	20 (13)	0.46
Cholangitis	4 (1)	4 (2)	0 (0)	0.189	2 (1)	2 (1)	1.00
Vena cava thrombosis	2 (1)	1 (1)	1 (1)	1.000	1 (1)	1 (1)	1.00
Embolism	2 (1)	2 (1)	0 (0)	0.569	1 (1)	1 (1)	1.00
Multi organ failure	10 (3)	7 (4)	3 (2)	0.519	5 (4)	5 (3)	1.00
Cardiac	21 (7)	13 (8)	8 (6)	0.668	8 (6)	13 (8)	0.54
Pneumonia	15 (5)	7 (4)	8 (6)	0.688	9 (6)	6 (4)	0.44
Other pulmonal	22 (7)	13 (8)	9 (7)	0.861	9 (6)	13 (8)	0.71

Abbreviations: ALR, anatomic liver resection; NALR, non‐anatomic liver resection.

Table 3 shows details stratified for patients with and without cirrhosis. Cirrhotic patients after ALR had significantly longer post‐operative ICU stays (*p* = 0.02) and significantly longer post‐operative in‐hospital stays (*p* = 0.01) compared to cirrhotic patients after NALR. Complications were significantly more severe in cirrhotic and non‐cirrhotic patients after NALR (*p* < 0.001).

**TABLE 3:** Details surgery and morbidity stratified for patient with and without cirrhosis.

	Patients with cirrhosis				Patients without cirrhosis			
	All patients	ALR	NALR	*p*‐values	All patients	ALR	NALR	*p*‐values
	*N* = 158	*N* = 64	*N* = 94		*N* = 140	*N* = 100	*N* = 40	
Days on ICU, mean (SD)	3 (5)	4 (6)	2 (2)	0.017	5 (26)	5 (27)	5 (25)	0.991
Days in hospital post‐surgery, mean (SD)	13 (9)	16 (9)	12 (8)	0.007	17 (29)	17 (28)	16 (31)	0.866
Complications (Clavien Dindo), *n* (%)								
0	56 (36)	43 (67)	13 (14)	<0.001	73 (52)	68 (68)	5 (12)	<0.001
1	24 (15)	5 (8)	19 (20)		13 (9)	7 (7)	6 (15)	
2	24 (15)	4 (6)	20 (22)		19 (14)	7 (7)	12 (30)	
3	20 (13)	6 (9)	14 (15)		15 (11)	10 (10)	5 (12)	
4	22 (14)	1 (2)	21 (23)		16 (11)	7 (7)	9 (22)	
5	11 (7)	5 (8)	6 (6)		4 (3)	1 (1)	3 (8)	
Specific complications, *n* (%)								
Bilioma	10 (6)	7 (11)	3 (3)	0.103	9 (6)	8 (8)	1 (2)	0.414
Bleeding	11 (7)	4 (6)	7 (7)	1.000	10 (7)	6 (6)	4 (10)	0.641
Cardiac	13 (8)	7 (11)	6 (6)	0.467	8 (6)	6 (6)	2 (5)	1.000
Cava thrombosis	1 (1)	0 (0)	1 (1)	1.000	1 (1)	1 (1)	0 (0)	1.000
Cholangitis	2 (1)	2 (3)	0 (0)	0.317	2 (1)	2 (2)	0 (0)	0.910
Embolism	1 (1)	1 (2)	0 (0)	0.846	1 (1)	1 (1)	0 (0)	1.000
Liver abscess	4 (3)	4 (6)	0 (0)	0.052	1 (1)	1 (1)	0 (0)	1.000
Liver insufficiency	20 (13)	9 (14)	11 (12)	0.846	13 (9)	13 (13)	0 (0)	0.038
Multi organ failure	5 (3)	2 (3)	3 (3)	1.000	5 (4)	5 (5)	0 (0)	0.349
Other pulmonal	13 (8)	6 (9)	7 (7)	0.890	9 (6)	7 (7)	2 (5)	0.957
Pneumonia	6 (4)	2 (3)	4 (4)	1.000	9 (6)	5 (5)	4 (10)	0.479

Abbreviations: ALR, anatomic liver resection; NALR, non‐anatomic liver resection.

### 3.3 Recurrence free and overall survival according to the type of liver resection

Median follow up time was 52.8 months. Table 4 illustrates OS and RFS after ALR versus NALR as well as patients with versus without cirrhosis of the same cohort compared using univariate and multivariate cox regression models with calculated HR. There were no significant differences in OS and RFS rates concerning the resection type. Regarding the underlying liver disease, there were no significant differences in OS and RFS with and without cirrhosis (*p* = 0.82 for OS and *p* = 0.64 for RFS). However, further subgroup analyses showed no significantly different OS (*p* = 0.64 after ALR and *p* = 0.18 after NALR) and RFS (see Table 5) regarding resection type and presence of cirrhosis. NALR showed comparable RFS in case of cirrhosis compared to non‐cirrhotic patients (*p* = 0.15).

**TABLE 4:** Survival analyses—overall survival (OS) and recurrence free survival, median follow‐up time: 52.8 months (95% CI 43.43–59.4).

	Median OS months (95% CI)	Survival rates in % (95% CI)				Hazard ratio	Adjusted hazard ratio
		1 year	2‐year	3‐year	4‐year	(95% CI, *p*‐value)	
Type of resection							
ALR	33.3 (28.1, 47.8)	74.8 (68, 82)	61 (53, 70)	46 (38, 56)	39 (30, 49)	1.26 (0.93, 1.72; 0.14)	1.15 (0.72, 1.85; 0.56)
NALR	49.1 (38.3, 63.3)	85 (79, 92)	72 (64, 81)	59 (50, 69)	51 (42, 61)		
Cirrhosis							
Cirrhosis	43.2 (32.3, 60.8)	82 (76, 89)	70 (63, 78)	53 (45, 63)	46 (37, 57)	0.86 (0.63, 1.17; 0.33)	1.06 (0.64, 1.75, 0.82)
No cirrhosis	38 (28.6, 53.3)	76 (69, 84)	62 (54, 71)	52 (43, 62)	42 (34, 53)		

	Median RFS months (95% CI)	Recurrence free survival rates in % (95% CI)				Hazard ratio	Adjusted hazard ratio
		1 year	2‐year	3‐year	4‐year	(95% CI, *p*‐value)	
Type of resection							
ALR	29.2 (21.8, 36.8)	74 (67, 81)	57 (49, 66)	41 (33, 51)	31 (23, 40)	1.26 (0.95, 1.67; 0.10)	1.42 (0.92, 2.20; 0.11)
NALR	42.9 (32.2, 52.8)	84 (78, 91)	71 (64, 80)	55 (47, 65)	44 (35, 54)		
Cirrhosis							
Cirrhosis	34.9 (29.1, 45.8)	81 (75, 88)	67 (59, 75)	47 (39, 57)	37 (29, 47)	0.89 (0.67, 1.17; 0.40)	1.11 (0.70, 1.78; 0.64)
No cirrhosis	33.3 (25.6, 42.9)	76 (69, 84)	60 (52, 69)	48 (40, 58)	36 (28, 47)		

*Note*: A hazard ratio >1 indicates higher risk of event for patients with anatomic resection of cirrhosis. Adjusted hazard ratio considers the following variables: sex, centre, stage, tumour size, BMI, ASA classification, cirrhosis, portal hypertension, diabetes mellitus, cardiac comorbidities and type of resection.

Abbreviations: ALR, anatomic liver resection; NALR, non‐anatomic liver resection; RFS, recurrence free survival.

**TABLE 5:** Subgroup analysis–overall survival and recurrence free survival – cirrhosis versus no cirrhosis for anatomic and non‐anatomic resection.

	Median OS months (95% CI)	Survival rates in % (95% CI)				Hazard ratio	Adjusted hazard ratio
		1 year	2‐year	3‐year	4‐year	(95% CI, *p*‐value)	
Anatomic resection							
Cirrhosis	30.3 (25.7, 52.0)	76 (66, 88)	64 (52, 79)	39 (26, 58)	36 (23, 55)	1.1 (0.71, 1.68; 0.68)	1.08 (0.59, 2.37; 0.64)
No cirrhosis	36.9 (21.8, 55.5)	74 (66, 84)	59 (50, 71)	50 (41, 63)	40 (30, 53)		
Non‐anatomic resection							
Cirrhosis	49.2 (38.9, 67.8)	87 (80, 94)	74 (65, 84)	61 (51, 73)	52 (41, 65)	0.72 (0.44, 1.18; 0.20)	0.58 (0.26, 1.28; 0.18)
No cirrhosis	42.9 (25.4, 64.3)	82 (70, 95)	67 (54, 85)	55 (40, 74)	47 (33, 68)		

	Median RFS months (95% CI)	Recurrence free survival rates in % (95% CI)				Hazard ratio	Adjusted hazard ratio
		1 year	2‐year	3‐year	4‐year	(95% CI, *p*‐value)	
Anatomic resection							
Cirrhosis	28.4 (21.2, 35.8)	72 (61, 85)	55 (43, 71)	31 (20, 48)	26 (16, 43)	1.15 (0.78, 1.71; 0.47)	01.31 (0.70, 2.48; 0.40)
No cirrhosis	33.3 (21.3, 44.9)	74 (66, 84)	57 (48, 69)	46 (37, 59)	33 (24, 46)		
Non‐anatomic resection							
Cirrhosis	45.0 (34.9, 57.5)	87 (80, 94)	74 (65, 84)	57 (47, 70)	44 (34, 58)	0.75 (0.48, 1.17; 0.21)	0.58 (0.28, 1.22; 0.15)
No cirrhosis	38.3 (25.1, 62.2)	79 (67, 93)	65 (52, 83)	51 (36, 70)	44 (30, 64)		

*Note*: A Hazard ratio >1 indicates higher risk of event for patients with cirrhosis. Adjusted hazard ratio consider the following variables: sex, centre, stage, tumour size, BMI, ASA classification, portal hypertension, diabetes mellitus, cardiac comorbidities.

**FIGURE 1:**
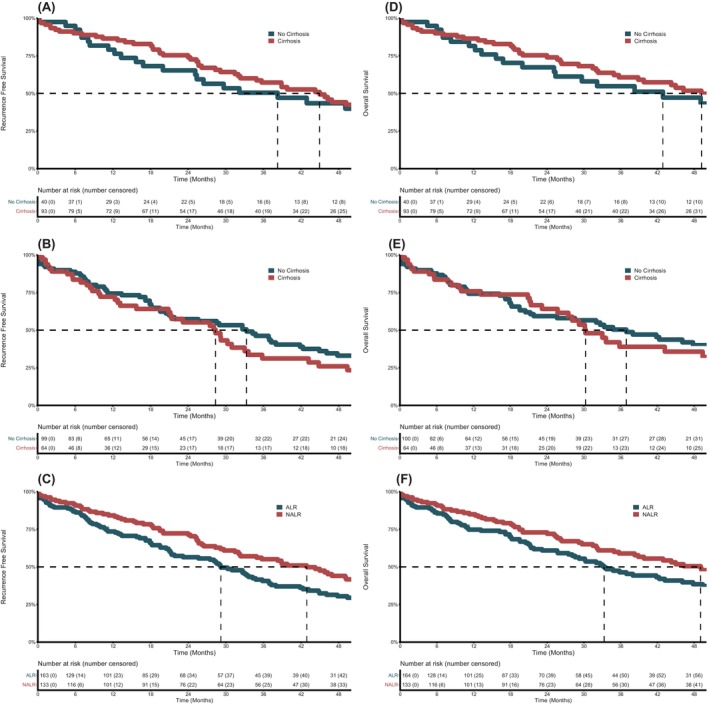
Overall survival curves and recurrence free survival curves. (A) Recurrence free survival in cirrhotic and non‐cirrhotic patients after NALR. (B) Recurrence free survival in cirrhotic and non‐cirrhotic patients after ALR. (C) Recurrence free survival after ALR and NALR. (D) Overall survival in cirrhotic and non‐cirrhotic patients after NALR. (E) Overall survival in cirrhotic and non‐cirrhotic patients after ALR. (F) Overall survival after ALR and NALR. ALR, anatomic liver resection; NALR, non‐anatomic liver resection.

Kaplan–Meier curves depicting RFS and OS according to the type of LR are shown in Figure 1. Patients undergoing NALR had no longer OS than patients undergoing ALR. Median OS of 49 months after NALR compared to ALR with a median OS of 33.3 months was statistically not significant (*p* = 0.56). A comparable non‐significant difference could be observed regarding RFS. Tumour recurrence occurred in 50% of the patients within 29 months after surgery in the ALR group compared to 43 months in the NALR group. Figure 1 also shows Kaplan–Meier curves depicting OS and RFS in cirrhotic compared to non‐cirrhotic patients, with no significant differences. Both comparisons indicate that patients with and without liver cirrhosis had a comparable OS and RFS after NALR compared to ALR.

## 4 CONCLUSIONS

To our knowledge, this is the first European multicentre study investigating ALR versus NALR for HCC in patients with and without cirrhosis. Our analyses showed no statistical significant differences for OS and RFS rates, also in the subgroup of patients with cirrhosis. However, post‐operative complications were significantly more frequent and severe after NALR, whereas liver specific complications showed no significant difference.

Currently, the benefits of ALR versus NALR for HCC are still discussed controversially with a lack of consistent evidence.^22^ Therefore, the present data support and especially for the European population expand previous results of other studies investigating OS, RFS and post‐operative complications after ALR versus NALR.^29^, ^30^

Due to the high incidence of recurrences, especially early recurrences within the first 2 years after LR for HCC (responsible for up to 70% of all tumour recurrences), ALR seems to have at least theoretical advantages compared with NALR.^31^ In particular, the multi‐centric pattern of newly developing HCC within a dysfunctional liver, as well as the high incidence of intrahepatic metastases originating from the resected tumour favour ALR.

Early recurrence is associated with tumour invasion into branches of the portal vein, leading to tumour thrombi in veins and new tumour lesions within the respective liver segments.^32^, ^33^ As a result, ALR could reduce the spread of tumour cells along the portal vein system. A retrospective Japanese analysis from 2020 showed that tumours with microportal vein invasion (vp1) were significantly more frequently associated with local recurrences after NALR compared to ALR.^34^ Still, this study did not show any impact of ALR on RFS or OS. While after ALR local recurrences in HCC with vp1 could be avoided, early recurrences occur also in vp0 HCC cases.^34^ The Japanese data showed excellent OS rate (90.9% 1‐year and 62.3% 5‐year OS after ALR and 91.8% 1‐year and 66.7% 5‐year OS after NALR), whereas our present European data showed as well no statistical differences between ALR and NALR for HCC but a 10%–20% lower OS rate compared with the Japanese data. Another retrospective study from 2023 observed that ALR could remove intrahepatic metastases within the resected segment, but had no impact on contralateral or extrahepatic recurrences.^35^

Especially in cirrhotic patients, NALR clearly has the advantage of preserving liver parenchyma, and therefore has the potential to reduce post‐operative liver failure. But it does not seem superior concerning avoidance of recurrences.^36^ These findings are consistent with our results. The majority of our patients with cirrhosis underwent NALR (70%), and liver insufficiency was not significantly different between the two groups in our collective. Interestingly, in our cohort cirrhosis was not associated with a negative impact on post‐operative liver failure as well as on OS and RFS, even if stratified for ALR and NALR.

A double‐blinded randomised controlled trial from China and Japan in 2017 showed that early recurrences occurred significantly less after ALR compared to NALR (30% vs. 59%).^29^ Nevertheless, RFS, OS and the occurrence of post‐operative or perioperative complications were similar between the ALR and NALR groups. This study was limited by its primary endpoint, defined as 2‐year local recurrence rate. The study group determined early recurrences as recurrences within the same segment as the primary tumour. Therefore, all extrahepatic recurrences as well as recurrences within another liver segment were excluded. Second, the majority of patients in this study population suffered from Hepatitis‐B induced chronic liver diseases, and the results may not be applicable to European patients with mostly other underlying liver diseases. Concerning the occurrence of post‐operative complications; however, the cited study is in line with our findings.

Since 2011, four meta‐analyses investigating ALR versus NALR have been published, mostly including retrospective studies.^23^, ^30^, ^37^, ^38^ Zhou et al. showed that ALR for HCC is associated with a significantly better 5‐year OS and less common local intrahepatic recurrences compared to NALR. In concordance with the present findings, mortality rate and perioperative complication rate were not significantly different between both groups.^30^ A meta‐analysis by Cucchetti et al. showed similar results, including most of the studies analysed by Zhou et al.^37^ Interestingly, a meta‐regression analysis suggested that OS and RFS depend on the underlying liver disease after ALR and NALR. The authors showed that cirrhotic patients, patients with severe liver dysfunction and patients with Hepatitis C infection more often underwent NALR instead of ALR. These three factors are known to be associated with higher recurrence rates after HCC resection.^39^ These data are in line with the present data, as patients with cirrhosis also received NALR significantly more often. In the meta‐regression, cirrhosis was associated with a significant difference in OS and RFS, suggesting that the observed differences in OS and RFS are mainly due to the presence of cirrhosis and rather than the type of resection. Additionally, a meta‐analysis from 2018 showed analogue results and similar 5‐year OS and RFS in both groups (ALR vs. NALR) in cirrhotic patients.^23^

In 2020, Jiao et al. conducted a meta‐analysis,^38^ which suggested advantages of ALR compared to NALR regarding 1‐, 3‐ and 5‐year OS and RFS in HCC patients. However, baseline characteristics among both groups including age, presence of cirrhosis, liver function and tumour size differed between both groups. The authors themselves indicated the heterogeneity, considering these limitations the results between both groups are not comparable.

More importantly, all of these meta‐analyses included mostly Asian studies, and only about 200 European patients were included in the meta‐analyses discussed above. As the underlying cause for cirrhosis and development of HCC is different across Asian and Western populations, results from Asian studies might not be applicable for Western populations. Our study presents one of the first European cohorts investigating the type of LR in patients with and without cirrhosis.

In our study, patients with liver cirrhosis and HCC more frequently underwent NALR. In addition, the rate of liver failure was not elevated after ALR. These findings support the results of other studies, which show that NALR reduces the risk of perioperative liver failure, especially in cirrhotic patients,^22^ while patients with sufficient liver function more often underwent ALR. Additionally, patients in both groups showed similar liver specific complication rates, despite the fact that patients with higher UICC stage more frequently received ALR with per se a higher risk for post‐operative complications. On one hand, a more extended LR has shown to be associated with a better oncological outcome,^16^ with ALR bearing the risk of increased complications.^40^ On the other hand, a meta‐analysis from 2018 including 12′429 patients has shown no differences between ALR versus NALR for HCC patients. The authors concluded that in appropriately selected patients both resection types are comparable concerning post‐operative mortality and morbidity.^23^ These results undermine the fact that resection type should be chosen according to liver function, tumour size and underlying liver disease.

In our cohort, 5% of patients (15/298) died within the first 30 post‐operative days. These results are more or less consistent with other studies showing a 30‐day mortality after LR for HCC below 5% in specialised clinics.^11^, ^14^, ^15^ Regarding the rate of post‐operative morbidity, the results are also consistent with the 30%–40% morbidity rates described in recent literature.^41^, ^42^, ^43^

In a multivariate Cox regression model, HR for OS and RFS was adjusted for age, sex, centre, UICC, tumour size, BMI, ASA, portal hypertension, cirrhosis, diabetes and cardiac comorbidities. Earlier studies have shown that these risk factors are commonly associated with poorer outcome after LR for HCC. Cirrhosis^44^, ^45^ as well as sex,^44^, ^46^ BMI,^47^, ^48^ age,^49^, ^50^, ^51^, ^52^ diabetes^49^ and tumour stage^53^, ^54^ could be shown to be independent risk factors for local recurrence and poorer survival. Interestingly, the present study shows no differences in OS and RFS between cirrhotic and non‐cirrhotic patients.

There are several limitations of our study. First, this is a retrospective and prospective non‐randomised study, which has inherent biases and does not allow any causal assumptions. Second, this study includes patients from Switzerland and Germany. Therefore, alcohol abuse and hepatitis C are the main etiological factors for cirrhosis, while within the Asian populations hepatitis B is a leading cause for cirrhosis. Moreover, in the United States and Europe, hepatitis C and alcohol abuse, together with non‐alcoholic fatty liver disease are also the main etiological factors for development of HCC.^55^, ^56^, ^57^, ^58^, ^59^

Ultimately, our study aims to fill the gap of needed results applicable for Western populations. There remains an unmet need of further prospective and randomised studies in Europe to investigate this very important point of interest for Hepato‐Pancreato‐Biliary Surgeons.

## AUTHOR CONTRIBUTIONS

**Jasmin Zeindler:** Conceptualization (equal); data curation (lead); formal analysis (lead); investigation (equal); methodology (equal); project administration (equal); writing – original draft (equal). **Gabriel Fridolin Hess:** Conceptualization (equal); data curation (lead); formal analysis (lead); investigation (equal); methodology (equal); project administration (equal); writing – original draft (lead). **Maximilian von Heesen:** Data curation (equal); formal analysis (equal); resources (supporting); writing – original draft (supporting). **Noa Aegerter:** Conceptualization (equal); investigation (equal); methodology (equal); visualization (equal); writing – review and editing (equal). **Cornelia Reber:** Conceptualization (equal); investigation (equal); methodology (equal); visualization (equal); writing – review and editing (equal). **Andreas Michael Schmitt:** Formal analysis (equal); project administration (equal); resources (equal); software (equal); validation (equal); writing – review and editing (equal). **Simone Muenst:** Conceptualization (equal); formal analysis (equal); methodology (supporting); resources (equal); validation (lead); writing – review and editing (lead). **Martin Bolli:** Formal analysis (equal); project administration (equal); writing – review and editing (equal). **Savas Deniz Soysal:** Conceptualization (lead); methodology (equal); supervision (equal); validation (equal); writing – review and editing (lead). **Otto Kollmar:** Conceptualization (lead); methodology (equal); supervision (equal); writing – review and editing (lead).

## ETHICS STATEMENT

Swiss ethics commission registration number: EKNZ 316/12. All patients included, were consented, in accordance with the ethics guidelines. Material from other sources: Not applicable.

## Data Availability

The data that support the findings of this study are available from the corresponding author (SDS), upon reasonable request. The data are not publicly available due to privacy or ethical restrictions.
